# Viewpoint: spinocerebellar ataxias as diseases of Purkinje cell dysfunction rather than Purkinje cell loss

**DOI:** 10.3389/fnmol.2023.1182431

**Published:** 2023-06-22

**Authors:** Josef P. Kapfhammer, Etsuko Shimobayashi

**Affiliations:** Institute of Anatomy, Department of Biomedicine, University of Basel, Basel, Switzerland

**Keywords:** cerebellum, neurodegenerative diseases, spinocerebellar ataxia (SCA), Purkinje cell, Protein Kinase C gamma

## Abstract

Spinocerebellar ataxias (SCAs) are a group of hereditary neurodegenerative diseases mostly affecting cerebellar Purkinje cells caused by a wide variety of different mutations. One subtype, SCA14, is caused by mutations of Protein Kinase C gamma (PKCγ), the dominant PKC isoform present in Purkinje cells. Mutations in the pathway in which PKCγ is active, i.e., in the regulation of calcium levels and calcium signaling in Purkinje cells, are the cause of several other variants of SCA. In SCA14, many of the observed mutations in the PKCγ gene were shown to increase the basal activity of PKCγ, raising the possibility that increased activity of PKCγ might be the cause of most forms of SCA14 and might also be involved in the pathogenesis of SCA in related subtypes. In this viewpoint and review article we will discuss the evidence for and against such a major role of PKCγ basal activity and will suggest a hypothesis of how PKCγ activity and the calcium signaling pathway may be involved in the pathogenesis of SCAs despite the different and sometimes opposing effects of mutations affecting these pathways. We will then widen the scope and propose a concept of SCA pathogenesis which is not primarily driven by cell death and loss of Purkinje cells but rather by dysfunction of Purkinje cells which are still present and alive in the cerebellum.

## Introduction

Spinocerebellar ataxias (SCAs) are neurodegenerative hereditary diseases affecting the cerebellum and presenting with typical symptoms and signs of cerebellar dysfunction, in particular a stance and gait ataxia often accompanied by nystagmus and varying additional problems (Klockgether et al., 2019; Muller, 2021). These diseases are rare with only 1–5 in 10.000 people affected (Bhandari et al., 2022) and these patients are then further stratified into more than 40 subtypes with different genes affected by the respective mutations. Nevertheless, there is remarkable interest into this disease from the scientific community (Soong and Morrison, 2018; Sullivan et al., 2019). Why are SCAs so interesting from a scientific point of view? One reason certainly is that the underlying mutations are by now well-defined and offer the chance to better understand the pathway from a defined genetic mutation to the disease manifestation. This is further helped by the finding that in most subtypes the cellular target are Purkinje cells, the major projection neuron of the cerebellar cortex. The connectivity pattern of the cerebellar neurons is very stereotyped and alterations can be identified relatively easy (Eccles, 1967). On the other hand, there is a remarkable heterogeneity of the affected genes, yet mutations in these different genes produce a rather similar pathology and disease phenotype. In this viewpoint article we will initially focus on one particular subtype, spinocerebellar ataxia type 14 (SCA14) (Chen et al., 2012), which is caused by mutations in the gene of Protein Kinase C gamma (PKCγ) and we will discuss how the mutations might cause the disease. In a second part we will widen the scope and propose a concept of SCA pathogenesis which is not primarily driven by cell death and loss of Purkinje cells but rather by Purkinje cell dysfunction.

### SCA14 mutations and possible pathogenic pathways

SCA14 (OMIM 605361) is a rather rare subtype of SCAs caused by mutations in PKCγ, a well characterized signaling protein kinase in Purkinje cells (Chen et al., 2012; Chelban et al., 2018). PKCγ is very strongly expressed in Purkinje cells and is involved in Purkinje cell neuronal plasticity, in particular in long term depression (LTD), the major form of adaptation of synaptic efficacy in cerebellar Purkinje cells (Ito, 2001; Watanave et al., 2022). While it was shown that PKCα can substitute for PKCγ for parallel fiber LTD expression (Leitges et al., 2004), there is good evidence that PKCγ is contributing to LTD induction (Shuvaev et al., 2011; Tsumagari et al., 2020). Furthermore, it is involved in developmental processes such as multiple climbing fibers (CFs) elimination (Saito and Shirai, 2002) and in Purkinje cell dendritic expansion (Kapfhammer, 2004).

The generation of PKCγ-deficient mice was reported in 1993 (Abeliovich et al., 1993). Studies with PKCγ deficient mice revealed that the innervation of CFs which are axonal projections from the inferior olivary nucleus to Purkinje cells was impaired, meaning that PKCγ is involved in multiple CFs elimination needed to establish a one-to-one innervation. Furthermore, PKCγ was shown to be involved in Purkinje cell dendritic expansion (Kano et al., 1995; Schrenk et al., 2002). However, PKCγ deficient mice showed no gross morphological changes in the cerebellum and in the Purkinje cells. Interestingly, PKCγ deficient mice show only a subtle phenotype and do not show typical signs of SCAs (Chen et al., 1995; Saito and Shirai, 2002) meaning that a mere loss of function of PKCγ is not sufficient for the development of SCA pathogenesis. The reason for this mild phenotype of loss of function mutations is probably found in the presence of other PKC isoforms which are also present in Purkinje cells, in particular PKCα, which is thought to compensate for the lack of PKCγ in Purkinje cells (Leitges et al., 2004).

As of today, more than 40 different mutations have been identified in the PKCγ gene from SCA14 patients. If one looks in which domains of PKCγ the mutations of human families affected by SCA14 are located, an intriguing pattern emerges. About 85–90% of the mutations cluster in the regulatory C1A and C1B domains of the protein which are binding diacylglycerol (DAG) and are required for relieving the inhibition of the kinase domain by the auto-inhibitory pseudosubstrate domain of the protein (Pilo et al., 2022). The remaining 10–15% of the mutations map to the kinase domain (Chelban et al., 2018; Schmitz-Hübsch et al., 2021). There is only little information about the neuropathology of human SCA14 cases, but Purkinje cell atrophy and loss have been found (Wong et al., 2018). Although the mutations are in the same protein, there is a huge range of phenotypes and a large variability in the degree of cerebellar atrophy (De Michele et al., 2022). These findings suggest that it is not a loss of function of PKCγ which is causative for the development of the SCA phenotype, but rather an altered regulation of the activity of PKCγ (Verbeek et al., 2005). This view is supported by studies looking at the kinase activity of mutated PKCγ. In a first systematic testing of the effects of the known mutations on PKCγ activity and function, it was shown that most of the SCA14 related mutants had an increased constitutive PKCγ activity in a cell-based assay (Adachi et al., 2008). These findings were confirmed in a recent study that found that basal activity was increased due to impaired kinase autoinhibition in all of the tested mutations causing SCA14 (Pilo et al., 2022).

These findings have raised the question whether an increased activity of PKCγ is causative for the development of an SCA14 phenotype. This question was addressed by two transgenic mouse models developed in our laboratory. In the first mouse model, the S361G mutation found in the human PKCγ kinase domain was transgenically expressed in Purkinje cells using a Purkinje cell-specific promotor construct (Ji et al., 2014). The mutated S361G-PKCγ was confirmed to have an increased constitutive activity in two studies using cell-based assays. In both studies, PKCγ activity was assessed by *in vitro* assays after transfection of the mutated PKCγ into COS7 cells, but not in Purkinje cells (Adachi et al., 2008; Pilo et al., 2022). The mouse model allowed the expression of the mutated S361G-PKCγ protein exclusively in Purkinje cells and offered the possibility to confirm the presence of increased biological PKCγ activity within Purkinje cells. From previous studies in our laboratory it is well known that increased PKC activity in Purkinje cells induced by treatment with phorbol esters during the phase of dendritic development compromises dendritic expansion and results in the development of a stunted dendritic tree of greatly reduced size in cerebellar slice cultures (Metzger and Kapfhammer, 2000; Schrenk et al., 2002; Gugger et al., 2012). Remarkably, the same stunted dendritic trees were found in Purkinje cells of cerebellar slice cultures derived from the S361G-PKCγ mice without pharmacological stimulation of PKC activity demonstrating the presence of increased PKCγ activity in the Purkinje cells and confirming the validity of the mouse model (Ji et al., 2014). Interestingly, these mice have a clear behavioral phenotype with deficits in motor coordination compatible with an SCA14-like pathology (Ji et al., 2014). The morphological changes found in these mice are subtler and there is no massive degeneration of Purkinje cells but a widespread reduction of Purkinje cell dendritic tree size and some localized loss of Purkinje cells in the cerebellum (Trzesniewski et al., 2019). Taken together, this mouse model shows that a constitutive activation of PKCγ in Purkinje cells results in an SCA14-like phenotype in the absence of widespread Purkinje cell loss.

There are two limitations of this mouse model: First, the phenotype is dependent on the activity of the Purkinje-cell specific L7 promoter and second the mutation is present in the kinase domain, not in the regulatory domains of the protein where the vast majority of SCA14 mutations were identified. We therefore investigated a second mouse model with a mutation in the pseudosubstrate domain (A24E) preventing the “closed” conformation of the PKCγ protein and forcing it permanently in the open active conformation (Pears et al., 1990).

The increased constitutive activity of PKCγ with such a mutation is known for a long time (Pears et al., 1990) but nothing was known about the consequences of the presence of such a mutation *in vivo*. A knock-in mouse carrying this A24E mutation was created and studied in our laboratory. As expected, Purkinje cells from A24E-PKCγ mice studied in cell culture developed only stunted dendritic trees which could be rescued by pharmacological inhibitors of PKC activity demonstrating the presence of increased constitutive PKCγ activity within the Purkinje cells (Shimobayashi and Kapfhammer, 2021). The increase of PKCγ activity could also be confirmed by an increased phosphorylation of PKC target proteins. The SCA14-like phenotype of these mice was similar to that of the S361G-PKCγ mice with a marked deficit in motor coordination and a reduction of Purkinje cell dendritic tree size. The heterozygous A24E also shows a clear motor deficit indicating that the heterozygous A24E is a valid mouse model related to SCA14 (Shimobayashi and Kapfhammer, 2021). Despite these clear indications of increased PKC activity, PKCγ protein expression was greatly reduced in homozygous A24E-PKCγ mice. This is in line with the nature of the mutation which fixes the protein in the open active conformation. In this open conformation the protein is subject to dephosphorylation and degradation giving it a rather short half-life and it is remarkable that despite this rapid degradation there is still an increased constitutive activity of PKCγ present in Purkinje cells of the A24E-PKCγ mice.

These two mouse models clearly show that the presence of an increased constitutive PKCγ activity in Purkinje cells does cause an SCA14-like pathology in the mice. While it can be taken for granted that most mutations have an increased basal activity, it is not clear whether this will result in an increased PKCγ activity present in the Purkinje cells *in vivo* as found in the two mouse models discussed above. There are several aspects about the mutated PKCγ proteins which may limit the impact of the increased basal activity. First of all, most of the mutated PKCγ proteins show a strongly increased tendency for aggregation and PKCγ aggregates are present in cells transfected with Wild type or mutated proteins (Seki et al., 2005, 2007; Takahashi et al., 2015). These aggregates were suggested to be neurotoxic and may contribute to Purkinje cell degeneration (Nakazono et al., 2018). While the presence and significance of PKCγ protein aggregates is controversial (Seki et al., 2009) and may vary from mutation to mutation, it is clear that PKCγ aggregates of mutated proteins can contribute to SCA14 pathology (Seki et al., 2005; Jezierska et al., 2014; Wong et al., 2018). Another important aspect for considering the role of PKCγ activity is the ability of the mutated proteins to translocate to the membrane in order to be fully activated by phosphatidylserine and DAG released at the plasma membrane. There is evidence that the translocation to the plasma membrane of mutated PKCγ proteins can be enhanced or impaired (Verbeek et al., 2008; Wong et al., 2018). In a cell-based assay, Adachi et al. (2008) found that two mutant PKCγs failed to phosphorylate one of PKCγ natural substrates, the Transient Receptor Potential cation channel subfamily C member 3 (TRPC3) channel (Adachi et al., 2008). Similarly, C1B domain mutations were found to have altered membrane dynamics and failed to activate downstream targets (Verbeek et al., 2008). The V138E mutation was shown to have a reduced availability in the cytoplasm and was directed to the insoluble fraction reducing their activity and possibly giving rise to protein aggregates (Jezierska et al., 2014). Altogether, mutated PKCγs show altered kinetics for the translocation to the membrane upon activation resulting in improper PKCγ signal transduction.

We have tested the potential of several mutant PKCγs to induce an increased PKCγ activity strong enough for showing a dendritic phenotype in developing Purkinje cells in dissociated Purkinje cell cultures as we had observed for Purkinje cells from S361G-PKCγ mice. When we transfected Purkinje cells with mutations either in the catalytic domain or in the regulatory domains, only the mutations in the catalytic domain could induce a dendritic phenotype (Shimobayashi and Kapfhammer, 2017). In later experiments, a strong dendritic reduction was also found with mutations in the pseudosubstrate domain (A24E, A24T). The A24T mutation was identified in human SCA14 patients (Chelban et al., 2018). The finding that the mutations in the regulatory C1A nor C1B domain did not induce a dendritic reduction of the Purkinje cells means that despite the observed increase in basal activity these mutations do not induce a long-lived and substantial increase of PKCγ activity in developing Purkinje cells. This is compatible with the failure of these mutations to induce an increased phosphorylation of their substrates (see above). Another important aspect considering the role of PKCγ activity for the pathogenesis of SCA is the presence of a nonsense mutation giving rise to a truncated PKCγ protein (Shirafuji et al., 2019). For this mutation, a dominant negative mechanism suppressing PKC activity is possible but it is difficult to conceive a mechanism involving an increase in PKC activity. The results of these studies suggest that both an increase of PKCγ activity and a reduction or alteration of PKCγ activity might cause the SCA14 phenotype.

### Other SCA mutations affecting Ca^2+^ homeostasis and synaptic function in Purkinje cells

The SCA14 mutation is affecting PKCγ which is part of a signaling cascade, the metabotropic glutamate receptor 1 (mGluR1)-PKCγ signaling pathway generating an intracellular calcium signal, which is crucial for changes in synaptic transmission and synaptic efficacy (Ito, 2001; Watanabe and Kano, 2011; Sugawara et al., 2017). The importance of this pathway with respect to SCA pathogenesis is underlined by the fact that molecules both upstream and downstream of PKCγ are also causative genes of SCAs. This is true for mutations in the mGluR1 receptor upstream of PKCγ (SCA44) (Watson et al., 2017), the Inositol 1,4,5-trisphosphate Receptor (IP3R1) upstream of PKCγ (SCA15, SCA29) (Tada et al., 2016), and the TRPC3 channel (SCA41) (Fogel et al., 2015) downstream of PKCγ. In all these cases the mGluR1-PKCγ signaling pathway will be affected and it will eventually be rendered dysfunctional by mutations in critical proteins (Shimobayashi and Kapfhammer, 2018).

Besides mutations which directly affect molecules in this signaling pathway, there is evidence that this pathway may also be altered in forms of SCAs caused by CAG repeats in the Ataxin genes. In SCA1 mutant mice, there is evidence that alterations of the mGluR1 signaling pathway contribute to pathogenesis (Power et al., 2016; Shuvaev et al., 2017). Similarly, there is evidence that altered activity of the mGluR1 signaling pathway is also contributing to pathogenesis in SCA2 (Meera et al., 2017) and SCA3 (Konno et al., 2014), indicating an important role of mGluR1 signaling for the pathogenesis of SCAs (Wu and Kapfhammer, 2021). Moreover, PKCγ expression and activity is altered in SCA1 and SCA2 suggesting the function of this signaling pathway is altered in these SCA subtypes (Chopra et al., 2018). In SCA5, mutations are present in β-III-spectrin resulting in abnormal formation and function of Purkinje cell synapses (Ikeda et al., 2006). Mutant β-III-spectrin decreases mGluR1 localization at dendritic spines of Purkinje cells suggesting that β-III-spectrin is directly involved in the mGluR1 signaling pathway (Armbrust et al., 2014).

Apart from mutations directly affecting the mGluR1-PKCγ signaling pathway, there are a number of mutations present in different SCA subtypes which do affect either ion channels or other molecules related to Purkinje cell synaptic function. A mutation affecting the cytoskeleton is found in SCA11 where Tau tubulin kinase is affected which is critical for ciliogenesis (Houlden et al., 2007; Bowie et al., 2018). Due to the mutations, cilia are lost in neurons and the synaptic connectivity and function of Purkinje cells is altered including changes of mGluR1 signaling and calcium homeostasis (Bowie et al., 2018). In SCA27, mutations are found in FGF14 which is regulating synaptic transmission and Purkinje cell spiking activity (Shakkottai et al., 2009; Yan et al., 2013). SCA13 is caused by mutations in KCNC3 encoding a potassium channel (Kv3.3) (Waters and Pulst, 2008). Although it is not completely clear how these mutations cause the SCA phenotype there is evidence that the mutations will eventually affect synaptic transmission in Purkinje cells (Waters et al., 2006; Zhang and Kaczmarek, 2016). Similar considerations apply to SCA19 and SCA22 which are caused by mutations in the KCND3 gene encoding voltage-gated potassium channel Kv4.3. Again, the exact consequences of the mutations on Purkinje cell synaptic transmission are not known, but there is evidence that the mutations do affect spiking patterns in Purkinje cells (Duarri et al., 2012) and may interfere with mGluR1 signaling (Ishibashi et al., 2016).

It is striking that of the various mutations causing SCAs a large part of them affects synaptic function and spiking response characteristics of Purkinje cells (Shakkottai et al., 2011; Dell’Orco et al., 2017), mostly by altering Purkinje cell calcium homeostasis and modifying the mGluR1 signaling pathway (reviewed in Robinson et al., 2020). This evidence establishes functional changes and altered response characteristics of Purkinje cells as one major mechanism underlying the pathogenesis of SCAs. For example, in the SCA1 mouse model, neuronal atrophy was found to be an adaptive change restoring repetitive firing (Dell'Orco et al., 2015). In an SCA6 mouse model, firing precision of floccular Purkinje cells was reduced resulting in a deficit of the vestibulo-ocular reflex VOR (Chang et al., 2022). Watanave and colleagues showed that in PKCγ deficient Purkinje cells, climbing fiber LTD was impaired (Watanave et al., 2022).

It is remarkable that many functional deficits associated with SCA are independent of Purkinje cell degeneration and cell death and are rather the consequence of Purkinje cell dysfunction. It should be noted that this type of pathogenesis affected by signaling molecules from mGluR1 to PKCγ may not be applicable to all cases of SCAs. Some mutations also affect mitochondrial function as in SCA28. It is known that SCA28 is caused by mutations in the mitochondrial protease AFG3L2 which leads to mitochondrial dysfunction (Maltecca et al., 2015). The brain activity needs lots of energy and mitochondria are the organelles generating the energy for cell activities, thus mitochondrial dysfunction may cause SCA more by apoptosis and loss of Purkinje cells rather than by Purkinje cell dysfunction (Harmuth et al., 2018; Barbier et al., 2022). For the rather frequent SCA cases caused by enhanced CAG repeats in the Ataxin transcriptional regulators it is open which type of pathogenesis is more important.

### A hypothesis for SCA pathogenesis with SCA14 as a starting point

While SCA14 is a rather rare subtype of SCA it falls into a group of SCA causing mutations which are affecting the calcium equilibrium in Purkinje cells. Calcium signaling in Purkinje cells is absolutely crucial for Purkinje cell functions (Kitamura and Kano, 2013). All of the known mechanisms modifying Purkinje cell functional activity do depend on calcium signaling, most notably LTD. Functional deficits of LTD may be an important contributor to cerebellar symptoms in SCAs (Tempia and Konnerth, 1994; Mitoma et al., 2022). A crucial aspect about calcium concentrations in Purkinje cells is that they can change rapidly upon external and internal signals (Kitamura and Hausser, 2011). For SCA14, there is some discussion of whether the mutations will cause a gain of function, loss of function or dominant negative phenotype (Wong et al., 2018). As discussed above, there is good evidence that all three types of phenotypes may be present with different SCA14 subtypes which may either cause an increased or a decreased level of PKCγ activity. This wide range of functional changes of PKCγ activity may look confusing first but becomes more meaningful when one considers the functional requirements for this signaling pathway of the Purkinje cells. For the proper function of these neurons it is essential that PKCγ activity can rise and fall fast, depending on external signals. What in the end matters for the cell is not whether the basic level of PKCγ activity is increased or decreased, what matters is the ability of the Purkinje cells to change its activity quickly and appropriately in order to produce a meaningful reaction to external signals. Any mutation which interferes with the dynamics of PKC activity or changes the magnitude and time frame of Purkinje cell responses will prevent Purkinje cells from functioning properly. In that sense, a constitutive activation of PKC activity as seen in the mouse models S361G-PKCγ mice and A24E-PKCγ mice may be “gain of function” with respect to PKC activity, but is a “loss of function” with respect to the dynamics and size of PKC activity changes upon external stimuli and it will interfere with the proper function of the cell. This broader concept could also explain the apparent discrepancy that a deletion of the PKCγ gene is not causing much functional deficits, but that a point mutation in the regulatory domain causes an SCA phenotype. In the case of the gene knockout, PKCγ function can be taken over by PKCα, and this will mostly preserve the dynamics and size of the response. But a mutation in the regulatory domain of PKCγ protein will change the dynamics and/or the amount of PKCγ activity in response to external signals, and because PKCγ is the dominant isoform in Purkinje cells and the PKCγ signal is dominant in Purkinje cells (Takahashi et al., 2017), an altered PKCγ activity will render the cell dysfunctional despite intact PKCα. Similar considerations apply for the mutations in pathways affecting the calcium equilibrium and synaptic function of the Purkinje cells (Hoxha et al., 2018). Because of the high spiking activity and of the strong stimulation by parallel and climbing fibers, Purkinje cells are strongly depending on an intact and efficient regulation of their intracellular calcium levels. Every mutation which is interfering with or modulating the calcium regulation will also alter the response characteristics of the Purkinje cells making them in many cases inappropriate. These mechanisms will make Purkinje cells dysfunctional, eventually producing an SCA pathology.

In our view, the labels “loss of function,” “gain of function” or “toxic gain of function” are too simplistic to properly describe the alterations caused by the mutations causing SCA which may affect Purkinje cell functional properties in multiple ways. The crucial aspect is a change in the response characteristics of Purkinje cells to external stimuli, eventually making the responses inappropriate and rendering Purkinje cells dysfunctional. The focus in SCA research in the past may have been too strongly on Purkinje cell loss and cerebellar atrophy (Durr, 2010; Reetz et al., 2013; Mascalchi et al., 2014). We think that in many cases Purkinje cell loss and cerebellar atrophy are just late manifestations of a Purkinje cell dysfunction going on for a much longer time period. In fact, there is considerable variability in the amount of cerebellar atrophy and Purkinje cell loss present in human SCA subtypes (Schulz et al., 2010; Goel et al., 2011) and the correlation of these findings with the clinical severity of the disease is poor (Robinson et al., 2020). Furthermore, from mouse studies, it is well known that a severe ataxia and motor impairment can be present in the absence of any or only little Purkinje cell loss (Clark et al., 1997; Shakkottai et al., 2004; Duvick et al., 2010; Hourez et al., 2011; Shakkottai et al., 2011) and that on the other side a substantial loss of Purkinje cells is compatible with near normal cerebellar function as long as there is a sufficient number (10–25%) of normal functioning Purkinje cells preserved (Martin et al., 2003, 2004).

**Figure 1:**
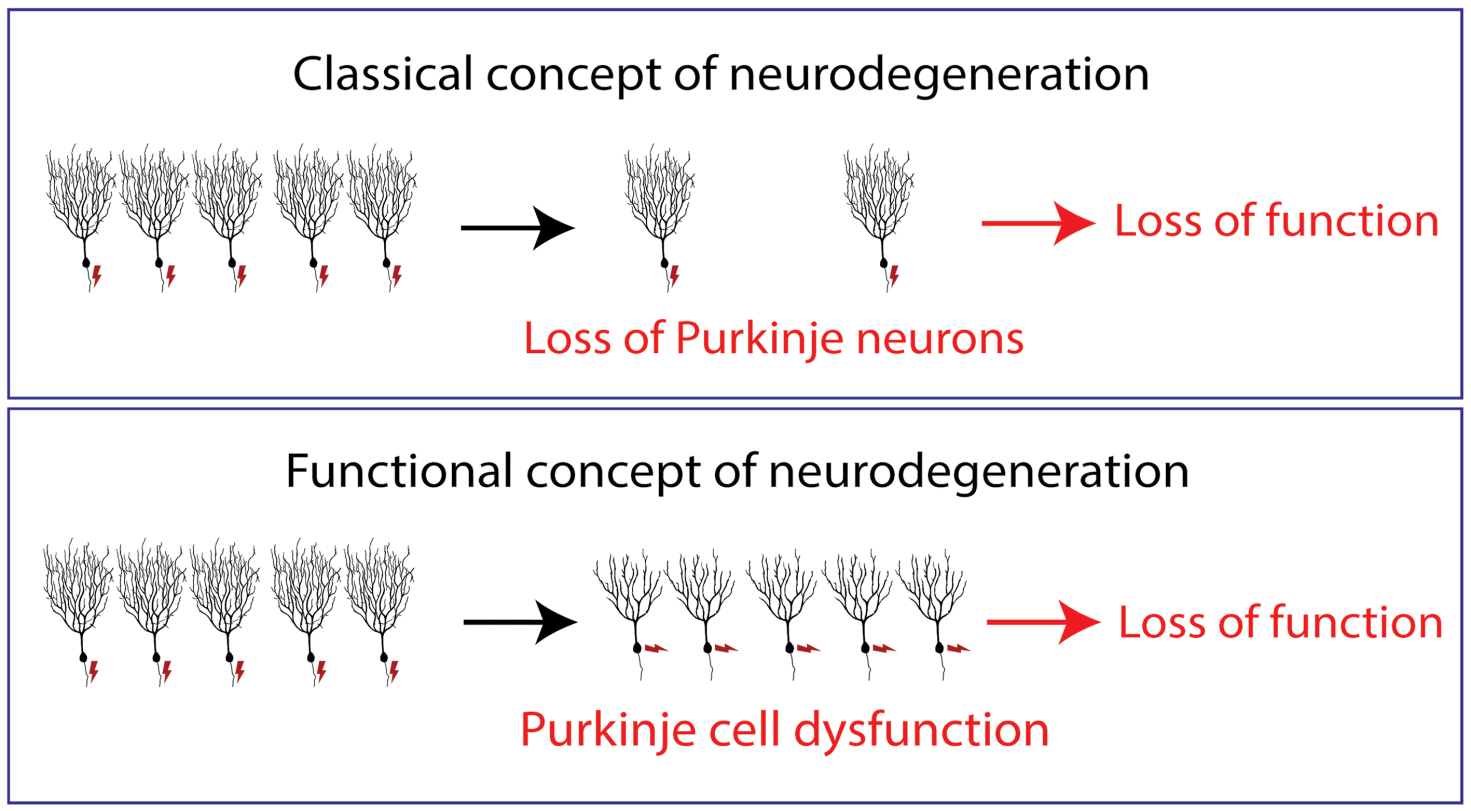
Normally neurodegenerative diseases are regarded as diseases caused by the death and loss of neurons. The symptoms of the patients in this view are caused by fact that not enough neurons are present to fulfill their tasks properly. In the concept of “Functional neurodegeneration” proposed in this article, the symptoms of the patients are caused by neurons which are alive but do not function properly, i.e., they may respond inappropriately to afferent stimuli (“dysfunctional neurons”). In this model, neuronal death is just a late event and not causally linked to the symptoms of the patients.

In many cases of human SCAs, the presence of cerebellar atrophy and Purkinje cell loss might be considered simply late signs of a dysfunctional cerebellum, but many of the functional deficits found in SCA patients are likely to be caused not by the Purkinje cells lost in the course of the disease but rather by the Purkinje cells which are still alive and present in the cerebellum of these patients, but are dysfunctional (Jayabal et al., 2015; Brown et al., 2018; Chang et al., 2022). We are convinced that a more appropriate view of SCAs is to see at least several subtypes in this group of diseases less as neurodegenerative in the classical sense, i.e., caused by the loss of neurons, but rather as diseases of Purkinje cell dysfunction (see Figure 1). Such a functional view of neurodegeneration may also apply to many other neurodegenerative diseases and is certainly an important aspect of functional deficits found in neurodegenerative diseases. For the patients, this may be good and bad news. On the one hand it means that the cause and type of Purkinje cell dysfunction may be different from subtype to subtype and may require quite different treatment strategies. On the other hand, it may give also hope because it means that in many cases of SCA there are still enough Purkinje cells present to principally ensure better cerebellar function if it was possible to rectify Purkinje cell activity by appropriate treatments.

## Author contributions

JK and ES developed the idea and wrote the manuscript. All authors were involved in discussions on the final manuscript.

## Funding

The authors are funded by the Swiss National Science Foundation, grant number 310030_189083.

## Conflict of interest

The authors declare that the research was conducted in the absence of any commercial or financial relationships that could be construed as a potential conflict of interest.

## Publisher’s note

All claims expressed in this article are solely those of the authors and do not necessarily represent those of their affiliated organizations, or those of the publisher, the editors and the reviewers. Any product that may be evaluated in this article, or claim that may be made by its manufacturer, is not guaranteed or endorsed by the publisher.
